# 

**Published:** 1883-03

**Authors:** 


					﻿Whole'Number,
CCOQLVIIL^
March, 1883.
Number 3,
Vol. XLVI.
TZEHZIE
CHICAGO
MEDICAL
OURNAL & EXAMINER.
CHICAGO:	*
PUBLISHED BY THE CHICAGO MEDICAL PRESS ASSOCIATION
188 Clark Street.
tg*Read the Notice of this Journal among Advertisements.
				

